# Effects of Various Muscle Disuse States and Countermeasures on Muscle Molecular Signaling

**DOI:** 10.3390/ijms23010468

**Published:** 2021-12-31

**Authors:** Kristina Sharlo, Sergey A. Tyganov, Elena Tomilovskaya, Daniil V. Popov, Alina A. Saveko, Boris S. Shenkman

**Affiliations:** Institute of Biomedical Problems RAS, Moscow 123007, Russia; sentackle@yandex.ru (S.A.T.); finegold@yandex.ru (E.T.); danil-popov@yandex.ru (D.V.P.); asaveko@gmail.com (A.A.S.); bshenkman@mail.ru (B.S.S.)

**Keywords:** skeletal muscle, disuse, unloading, atrophy, disuse countermeasures, protein synthesis, protein breakdown, myosin phenotype, oxidative capacity

## Abstract

Skeletal muscle is capable of changing its structural parameters, metabolic rate and functional characteristics within a wide range when adapting to various loading regimens and states of the organism. Prolonged muscle inactivation leads to serious negative consequences that affect the quality of life and work capacity of people. This review examines various conditions that lead to decreased levels of muscle loading and activity and describes the key molecular mechanisms of muscle responses to these conditions. It also details the theoretical foundations of various methods preventing adverse muscle changes caused by decreased motor activity and describes these methods. A number of recent studies presented in this review make it possible to determine the molecular basis of the countermeasure methods used in rehabilitation and space medicine for many years, as well as to identify promising new approaches to rehabilitation and to form a holistic understanding of the mechanisms of gravity force control over the muscular system.

## 1. Introduction

Skeletal muscle is an extremely flexible organ, since it undergoes various structural and functional changes in response to external and internal stimuli. The key regulatory signal for muscles comes from muscle activity, i.e., the level and duration of the muscle loading [1]. The adaptation of muscles to increased load has been the subject of interest since ancient times; however, much less attention has been paid to muscle adaptation to a reduced load or to complete inactivity. In recent decades, the development of scientific and technological progress has led to a change in lifestyle and a decrease in the motor activity of a huge number of people. The progress in medicine has made it possible to recover from severe injuries, often resulting in prolonged confinement to bed. The inactivation of skeletal muscles is often a side effect of the treatment of various diseases that require prolonged bed rest, which can complicate the rehabilitation of patients [2]. Nowadays it is obvious that a person’s health and well-being directly depend on the state of their skeletal muscles, which affects the functioning of the endocrine, nervous, cardiovascular and immune systems [3]. Therefore, the development of countermeasures against changes that occur in skeletal muscles as a result of their decreased activity (functional unloading) is important for human health. This review is devoted to the description of the main changes in disused skeletal muscles (cast immobilization, head-down tilt bed rest, “dry” immersion, space flight and various animal models) and countermeasures against changes occurring under these conditions, including both widely used approaches and those just starting to be put into practice.

According to their functions, skeletal muscles can be divided into two types: locomotor muscles, which are primarily responsible for voluntary locomotions, and “tonic” (postural) muscles, which play a key role in maintaining posture and resisting gravity [4]. These two types of muscles differ in the type of work they perform (primarily in the pattern of neuronal impulses and in the duration of work per day), and in the relative proportion of fibers of different types (“fast-twitch” or “slow-twitch”), which determines their differences in the type of metabolism and contractile activity. Moreover, the properties of fibers of the same type differ significantly depending on whether they belong to a predominantly “locomotor” muscle or a predominantly “tonic” muscle [5]. Data on different models of muscle inactivation that have been accumulated since the 1960s show that tonic and locomotor muscles have different sensitivities to various types of decreases in contractile activity. Postural muscles, predominantly consisting of muscle fibers of the “slow-twitch” type, are the most vulnerable to conditions of disuse and support elimination. The most complete elimination of the support stimulus is observed under space flight conditions, in the model of hindlimb unloading in rodents and unilateral lower limb suspension (ULLS) or “dry” immersion in humans; some weakening of the support stimulus for the muscles of the lower limbs is observed in bed rest as well. In contrast, the locomotor muscles undergo atrophy under when there is a decrease in mechanical loading, even if the support stimulus is present (the most typical model of such an effect is a restricted activity model for humans or animals).

**Figure 1:**
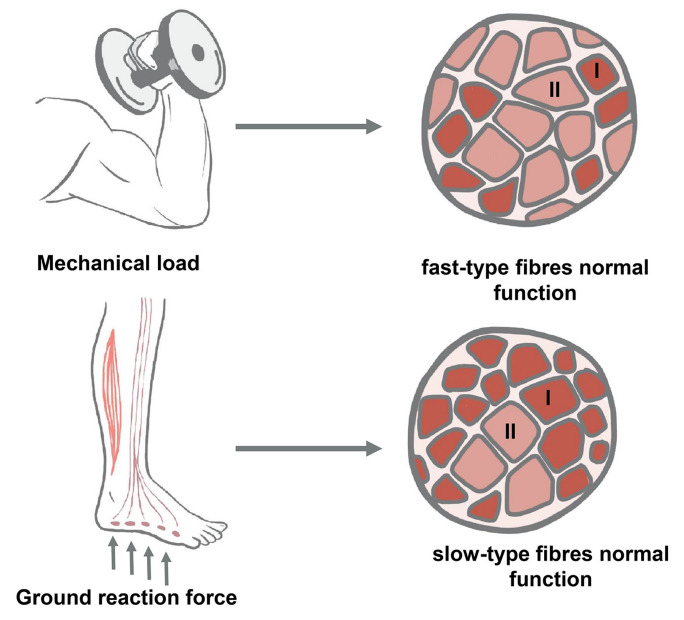
Involvement of mechanical load and ground reaction force into the regulation of slow-type (type I) or fast-type (type II) skeletal muscle fibers.

In real-life clinical practice, the conditions of muscle inactivation usually combine both the elimination of support and the elimination of mechanical loading, affecting both locomotor and tonic muscles. Therefore, when developing countermeasures against changes caused by some form of decrease in muscle contractile activity, it is necessary to take into account both the ratio of supportlessness and the decrease in mechanical loading in a particular model, and assess the involvement of tonic and locomotor muscles in negative changes separately (Figure 1).

## 2. Models of Decreased Activity of Skeletal Muscles and Their Functional Consequences

Currently, the decreased activity of skeletal muscles is studied in a wide range of models which differ significantly both in effects and in the dynamics and degree of adverse changes manifested. Key research models are head-down tilt bed rest (HDT BR), immobilization (with a cast or leg brace), step reduction, space flight, “dry” immersion and hindlimb unloading [6].

Head-down tilt bed rest is one of the most popular models used for studying the functional unloading of skeletal muscle in humans. HDT BR with a 6-degree tilt is a model for studying the chronic atrophic and adaptive effects of the functional unloading of the skeletal muscles [7]. The effects of HDT BR include both a decrease in the mechanical loading of muscles and a partial elimination of the support stimulus due to the redistribution of the support reaction force from the soles of the feet to the posterior surface of the human body. Atrophy of different muscles with different contents of fast-twitch and slow-twitch fibers was documented for HDT BR. Many studies have shown that HDT BR leads to a significant reduction in muscle strength and mass. A 60-day HDT BR led to a decrease in the cross-sectional area (CSA) of the lower back muscles, whereas a 120-day HDT BR to a decrease in the CSA of the soleus muscle; in both cases, the atrophy correlated with a decrease in the contraction strength of these muscles [8,9]. After 84-day HDT BR, magnetic resonance imaging showed a 17% reduction in mass and a 40% reduction in the strength of the lateral femoris muscle [10]. A different 90-day experiment showed a 26% decrease in the CSA of the lower leg muscles, as well as an 8% decrease in the CSA of the forearm muscles [11]. Moreover, similar results were obtained in a 35-day experiment, in which the CSA of the “slow-twitch” fibers of the lateral femoris muscle decreased to a significantly higher degree than the CSA of the “fast-twitch” fibers of the same muscle [12]. Short-duration HDT BR (17–20 days) also resulted in a 10–12% decrease in the CSA of the lower leg muscles [13]. On the other hand, it was also shown that not all muscles of the lower limbs are affected by prolonged HDT BR; in particular, after a case of 60-day HDT BR, there was no significant decrease in the volume of the rectus femoris, adductor brevis, gracilis or pectineus muscles in comparison with their state before the experiment [14].

The immobilization of limbs makes it possible to study the local effect of a decrease in motor activity on various muscle groups, as well as to identify the consequences of muscle immobilization in traumatology. The immobilized muscles of the lower limbs also exhibit the effect of eliminating the support stimulus on the soles of the feet (when the limb function is disabled). One of these models is limb immobilization using a cast or a special cuff that fixes the limb. A two-week cast immobilization leads to a decrease in the mass, CSA and strength of the quadriceps femoris muscle by 9%, 5–8% and 23%, respectively [15,16]. A 6-week cast immobilization of the lower limb muscles leads to the atrophy of the soleus muscle (19%), as well as the tibialis (10.7%), gastrocnemius medialis (23%) and gastrocnemius lateralis (17%) muscles; the atrophy of the muscles of the contralateral leg is also observed, although to a much lesser extent. Shorter (5-day) periods of lower limb cast immobilization led to a similar decrease in muscle mass [17]. A 5-day study showed a 9% decrease in the strength and a 4% decrease in the CSA of the quadriceps femoris muscle [18]. Thus, in the model of cast immobilization, as well as in bed rest, both fast-twitch and slow-twitch skeletal muscles fibers are subjected to atrophy to a comparable extent.

Space flight exerts a unique influence on skeletal muscles, since under these conditions both the support reaction force and the direct effect of weight are eliminated, which cannot be achieved on Earth. Thus, the conditions of space flight can be called an “ideal” model for studying the role of support, reaction force and weight on the functioning of skeletal muscles. Numerous works devoted to studies of the influence of space flight conditions indicate a significant decrease in skeletal muscle mass, the CSA of muscle fibers and muscle strength [6]. The longer the stay in zero gravity, the more pronounced is the atrophy of the skeletal muscles; 115–197 days of weightlessness lead to a 20% decrease in the mass of the soleus and gastrocnemius muscles and a 10% decrease in the mass of the quadriceps of the thigh [19]. Another study showed that a 180-day space flight significantly reduces the strength and size of the fibers of the soleus and gastrocnemius muscles. In addition, that study showed that the degree of atrophy depends on the type of muscle fibers. For example, the fibers that were the most susceptible to atrophy were the “slow-twitch” postural fibers of the soleus muscle. Furthermore, the fibers most resistant to microgravity conditions were the “fast-twitch” fibers of the gastrocnemius muscle, which mainly perform the locomotor function [20]. Thus, prolonged or brief elimination of the effect of gravity leads to reduced weight bearing by postural muscles and a decrease in their contractile activity. A decrease in the activity of these muscles results in their atrophy, a decrease in their contractile properties, and growing muscle weakness. However, even under terrestrial conditions, there are ways to eliminate the support stimulus.

In the early 1970s, E.B. Shulzhenko and I.F. Will-Williams at the Institute of Biomedical Problems of RAS developed a method for the functional unloading of skeletal muscles which they named “dry immersion” (DI) [21]. This method makes it possible to study the influence of negligible values of the support reaction force on the human motor system and to simulate a number of effects of space flight under terrestrial conditions. The volunteer lies down on a waterproof fabric placed in a bathtub and is thus submerged under the water to the level of the neck, with the fabric area significantly exceeding the surface area of the water. Excess fabric allows the subject’s body to be submerged from all sides [22]. This model makes it possible to simulate the effects of weightlessness, since the force of gravity in this case is balanced by the buoyant force. Thus, DI reproduces three effects of weightlessness: the decrease in muscle activity, the elimination of support signals and the elimination of the vertical distribution of the fluid in the blood vessels [22,23]. Moreover, under the conditions of this model (as well as under the conditions of space flight, especially for animals placed in tight compartments) there is also a limitation of muscle mobility. Staying under conditions of “dry” immersion for 7 days results in a number of changes at the cellular level in the human postural muscles [24,25,26]. Under these conditions, researchers observed a decrease in soleus muscle fiber diameter and the maximum value of the isometric force, calcium sensitivity and transverse stiffness, CSA of predominantly “slow-twitch”-type fibers, and of the relative content of titin, nebulin, desmin and α-actinin. These changes developed along with a decrease in the electrical activity of this muscle [27]. Earlier studies showed that as early as on the 3rd day of “dry” immersion, the slow-twitch motor units did not participate in the work of maintaining a small load [28]. It is interesting to note that the depth of atrophic changes that were found after 7 days of immersion is usually developed after longer periods of bed rest [29,30]. After 7 days of DI, a 24% decrease in the CSA of the slow-twitch fibers of the soleus muscle was detected, whereas there was no decrease in the CSA of the fast-twitch fibers of the same muscle [31]. Nevertheless, after 3 days of DI, a significant decrease in the CSA of the slow-twitch fibers of the vastus lateralis muscle of 7.5% and in the fast-twitch fibers of 8.9% was observed [23]. After 7 days of DI, a decrease in the CSA of slow-twitch and fast-twitch fibers of the vastus lateralis muscle had already reached 17.3% and 15.3%, respectively [23]. Under conditions of dry immersion, the contractile properties of the human soleus muscle were also studied. After 7-day DI, the maximum strength of the fibers of the soleus muscle decreased by 32% [26,31]. Similar data were obtained after 7-day DI and 7-day space flight using isokinetic dynamometry [32,33]. It should be noted that the decrease in the maximum strength of voluntary contraction in the DI experiment was more pronounced.

The most complete limitation of skeletal muscle mobility with the concomitant elimination of support afferentation occurs after various injuries affecting the nervous system, such as denervation of a muscle or muscle group, spinal cord injuries, various paralyses, as well as in intensive care units. In such conditions, both the afferent signal from the muscle and neuronal stimulation are absent. In some disorders, there is also no trophic effect of the nerve on the muscle fibers, which leads to catastrophic changes in both the fast-twitch and slow-twitch fibers of skeletal muscles [34,35,36]. In the case of life-threatening diseases (critical illness), muscle damage is also caused by the diseases themselves or their complications (sepsis, inflammation), as well as by drug therapy (for example, the use of glucocorticoids), and thus the data obtained in the study of such conditions cannot be used when considering the functional unloading of skeletal muscles. Therefore, a detailed description of the models associated with denervation is not included in this review.

There are also models studying a partial decrease in muscle activity with the full preservation of the support stimulus. One such model is step reduction (SR). In the SR experiment, subjects reduce the amount of daily walking (while counting each step with a pedometer) to the minimum values (750–5000 steps) [37,38]. The lower threshold for the number of steps (~750) approximately corresponds to the activity of outpatients [39]. The reduction of physical activity in the SR model to such low values does not lead to a complete functional unloading of skeletal muscles, but has serious physiological consequences [40]. Restriction of activity by 91% (from 13,054 to 1192 steps per day) reduces protein synthesis in skeletal muscle fibers by 22% [41]. The physiological consequences of even short periods of reduced activity in the SR model lead to a decrease in skeletal muscle strength and mass, as well as a decrease in insulin sensitivity. A reduction in the number of steps to less than 1500 per day for two weeks leads to a decrease in the maximum oxygen consumption [38] and a significant decrease in the maximum voluntary strength of the lower limbs [42,43].

Studies in animals (mainly in rodents and primates) also include models with the elimination of the support stimulus as the primary effect and models with the restriction of muscle contractile activity. At present, it is questionable whether animal reduced-activity models can be compared with human models. Nonetheless, the main functional and structural changes (muscle fiber atrophy, transformation of the phenotype, decrease in maximum strength), as well as changes at the molecular level (a decrease in protein synthesis and the level of mitochondrial biogenesis, an increase in the expression of proteolytic markers, changes in the expression pattern of myosin genes) are observed in both rodents and humans. Obviously, the dynamics of changes occurring during functional unloading of the muscles should differ in rodents and in humans. To date, there are insufficient data for most models and it is not possible to clearly compare the timing of the development of certain molecular responses to functional muscle unloading in different mammalian species. Nevertheless, when translating a number of results from animal models to humans, it is necessary to take into account the type of skeletal muscles, whether “tonic” or “locomotor”. A number of species of small animals, in particular mice (in contrast to rats), do not have purely “tonic” muscles at all, which imposes certain limitations on studies of the effects of supportlessness in these animals [44]. Rodents also have a type of myosin heavy-chain IIB isoform which is absent in humans [45].

The model of rodent hindlimb unloading is widely used to study muscle plasticity. One of the reasons this model is widely used is the similarity of many of its effects to those of space flight. It has been approved by many researchers as adequate for simulating microgravity, studying changes in skeletal muscle during space flight and the adaptation of skeletal muscles to decreased activity [46,47,48]. In this model, the animal is suspended by the tail in such a way that its hindlimbs do not touch the floor of the cage, whereas it is able to freely move around the cage. Thus, the support reaction force is eliminated.

In rat models of immobilization and hindlimb unloading, muscle loss is usually greater in the lower leg extensors (soleus, gastrocnemius muscles) than in flexors (extensor digitorum longus) [5,49,50]. A reduction in the CSA is observed first in slow-twitch, then in fast-twitch fibers of the IIa type, and then in IIx and IIb types [49,51], which is usually associated with elimination of the postural function in conditions of a supportless environment [52]. Atrophy of myofibrils under conditions of simulated hypogravity leads to a deterioration of the muscle contractile properties [53,54,55]. After 7-day hindlimb unloading, the relative weight of the soleus muscle decreased by 21% compared to the control, the maximum tetanic tension normalized to muscle weight was significantly reduced, and the contraction time decreased by 25% [56]. After 16-day hindlimb unloading, the absolute weight of the soleus muscle decreased by 50% and 65%, the fiber size decreased by 66% and the fiber length and the amount of sarcomeres decreased by 14% compared to control animals [57]. After 4-week hindlimb unloading, the weight of the unloaded soleus muscle was 42% lower than in control animals, whereas the strengths of maximum isometric contraction and tetanic tension were reduced by 62% and 69%.

The method of restricting the activity of animals while preserving the support stimulus is a partial limitation of mobility. In this model, the rodents are kept in small cages that restrict the movement of the animals. In [58], the weight of the soleus and plantaris muscles decreased by 10% and 27%, respectively, after 21 days in such cages. A decrease in the CSA was observed only for the plantar muscle, with the limitation of activity having a greater effect on the fast-twitch muscle fibers. These results differ from the earlier data obtained under the conditions of weightlessness or hindlimb unloading, which showed a greater atrophy of slow-twitch muscle fibers than fast-twitch ones [5,49]. In a recent experiment with a 21-day activity restriction in rodents, a decrease in the mass of both the soleus muscle and EDL was found; however, a decrease in protein synthesis and mTOR activity (determined by the level of phosphorylation of p70S6k) was observed only in the “fast-twitch” EDL [59]. Moreover, in connection with the problem of activity restriction, it is necessary to consider the data obtained on primates, when comparing the conditions of space flight and staying in the same capsule as those in the flight, but on Earth. Animals in space flight showed a significant decrease in the CSA of the vastus lateralis muscle, which was also observed in animals staying on Earth, whereas the sizes of the slow-twitch fibers of the soleus muscle decreased only under conditions of space flight [60,61]. These data demonstrate the different effects of supportlessness and mobility limitation for the locomotor and tonic components of the muscular system.

Cast immobilization can also be studied in animals. During immobilization, the joint of the hindlimb of the animal is fixed in the desired position using a cast bandage or needles. Experimental immobilization (with a needle) was used by Fischbach and Robbins [62]. They observed a significant reduction in impulse activity to 5–15% [62], <10% of the control [63], as well as a significant reduction in the strength of muscle contraction. Depending on the angle of fixation of the joint, the degree of muscle atrophy can be different, whereas the length of the muscle in a fixed state is very important [64]. A shortened muscle undergoes atrophy more strongly than a stretched muscle, and the decrease in functional activity in a shortened state is stronger [65,66,67]. Presumably, this effect is associated with the role of mechanical tension in the regulation of muscle mass.

Another model of the functional unloading of skeletal muscles of animals is tenotomy. In this model, a transection or partial dissection of the tendon is performed, which leads to a decrease in the mechanical loading and weakens the afferent response of the muscle due to the inactivation of muscle spindles. At the initial stages of tenotomy, in contrast to the hindlimb unloading model, the EMG activity of the tenotomized muscles of the hindlimbs is retained for at least one week [68,69]. After tenotomy, muscle weight and its strength characteristics decrease; atrophy occurs faster than in other models of functional unloading of the muscle. Weight loss can reach 50% or more. The weight of the soleus muscle of the rat after a 12-day tenotomy decreased approximately twofold [70,71]. After tenotomy, predominantly slow-twitch muscle fibers of tenatomized muscles undergo atrophy [72]. The key muscle inactivity models and their effects on slow-type and fast-type fibers are listed in Table 1.

## 3. Molecular Signaling Alterations in Skeletal Muscles under Disuse Conditions

The regulatory mechanisms involved in negative changes in the skeletal muscles under various disuse conditions have been studied for decades and researchers are still far from reaching a complete understanding. Since there are a lot of excellent and detailed reviews dedicated to various aspects of muscle signaling under muscle inactivation, this chapter of our review briefly describes the key molecular mechanisms determining muscle atrophy, fiber-type transition, as well as decreases in oxidative capacity and muscle functional characteristics.

The key signaling events of early muscle inactivity (caused by supportlessness or immobilization) are a dramatic decrease in energy consumption (ATP/ADP ratio increase, glycogen accumulation), calcium ion accumulation (accompanied by ROS production), a nitric oxide production decrease and the inactivation of mechanosensors [73,74,75]. These early changes cause an “intermediate” signaling state between actively working muscle (which is characterized by a low ATP/ADP ratio, high calcium and ROS levels, a high level of NO and active mechanosensors) and the resting muscle (high ATP/ADP ratio, low calcium and ROS levels, low NO and inactive mechanosensors). In this state, the negative effects of calcium/ROS accumulation cannot be compensated for by activity-induced defense mechanisms, so this leads to development of muscle atrophy and subsequent alterations. In the next chapters we describe the detailed regulatory mechanisms mediating the response of skeletal muscles to these changes.

It should be noted that the complete disappearance of postural soleus muscle EMG activity, observed during the first 1–3 days of muscle disuse, is followed by the restoration of EMG to control levels between the 6th and 9th days of rodent hindlimb suspension, and the further increase of EMG above the control level [76] A similar effect is observed in humans and animals after spinal cord injuries, although the time before the reappearance of EMG is longer [77]. In the hindlimb suspension model, the reappearance of EMG is accompanied by a decrease in the ATP/ADP ratio and activation of some activity-dependent signaling pathways (AMPK activation, NFAT entrance into muscle nuclei, myosin IIa expression restoration) after 14 days of rat hindlimb unloading [78,79]. It should be underlined that in the earlier stage of muscle unloading (1–3 days) AMPK is inactivated, the ATP/ADP ratio is high and the NFATc1 myonuclear content is twofold lower than that in the control [80,81,82]. However, these changes during later unloading stages do not lead to the restoration of muscle function. Moreover, some of these changes even enhance atrophy development [74]. These facts indicate that the time-course of signaling events during muscle disuse (especially the early stages of muscle disuse, 2 weeks and earlier) is not just a linear developing process, and the key mechanisms that determine atrophy development as well as other alterations are different at different time-points of disuse, even in the same disuse model and the same muscle. Nowadays the causes and effects of disuse-induced slow-tonic muscle activity are almost completely unclear; however, the presence of this activity, as well as its non-linear effect on muscle signaling, cannot be ignored when studying disused muscle functioning.

### 3.1. Effect of Reduced Motor Activity on Protein Synthesis in Skeletal Muscle

The loss of skeletal muscle mass (muscle atrophy) is caused by alterations of protein homeostasis in skeletal muscle fibers, i.e., by a decrease in the level of protein synthesis and/or by an increase in the rate of protein degradation. The relative contribution of these two processes to the atrophy caused by functional unloading depends on the studied model, as well as on the duration of the unloading. As early as at the 1st day of the hindlimb unloading of rodents, a significant decrease in the level of protein synthesis was observed in the soleus muscle [83]; at the same time, there was not yet any significant decrease in muscle mass observed. A reduced level of protein synthesis, determined using the SuNSET method, was also observed at later stages of hindlimb unloading (3rd, 7th, 14th days) [83,84,85]. A decrease in the level of protein synthesis assessed using radioactive labeling was observed after 3-, 4-, 7- and 28-day or hindlimb unloading of rodents [86,87,88,89].

A decrease in the level of protein synthesis as a result of the functional unloading of skeletal muscles was also found in humans; for example, Gibson et al. showed that people exposed to ULLS under fasting conditions had a ~30% lower protein synthesis rate compared to a contralateral non-immobilized limb [90]. Subsequent studies confirmed the decrease in protein synthesis, for example, a ~50% decrease in the synthesis rate was observed after 2-week HDT BR [91,92] and cast immobilization [15]. Later, Kortebein et al. reported a ~30% reduction in protein synthesis measured over a 24-h period in older adults after 10 days of bed rest [93].

Nowadays, the intensity of protein synthesis in mammalian skeletal muscles is believed to depend both on the efficiency of translation and on the translational capacity [94]. Translation efficiency is the rate of protein synthesis per ribosome, whereas translational capacity is determined by the amount of ribosomes per unit of tissue and, accordingly, depends on the amount of ribosomes in muscle fibers [94,95]. It should also be borne in mind that in order to fully understand the mechanisms that control protein synthesis in the skeletal muscles, it is necessary to take into account the duration of gravitational unloading [95,96].

The mTOR signaling protein (the mechanistic target of rapamycin) is the main center of the signaling network involved in the regulation of mRNA translation and thus translation efficiency [97]. Studies in vitro and on rodents show that this system responds to important stimuli responsible for the regulation of protein synthesis: (1) insulin and insulin-like growth factor-1 (IGF-1) via the IRS (insulin receptor substrate) and PI3K (phosphatidylinositol-3) signaling pathways, (2) amino acid levels via the leucyl-tRNA/Rag-mTORc1 pathways, (3) energy stress via the AMPK/eEF2 (AMP-activated protein kinase/eukaryotic elongation factor 2) pathway, and (4) mechanical stress via various mechanosensory molecules [97,98]. In conditions of normal activity in mammals, the IGF-1/Akt/mTORC1 signaling pathway acts as a regulator of translation initiation in the skeletal muscle [99,100]. This signaling pathway is initially activated via IGF-1 binding to its specific receptor (IGF-1R), which triggers a signaling cascade, resulting in the phosphorylation of the insulin receptor 1 substrate (IRS-1). Next, the phosphorylated IRS-1 binds to phosphatidylinositol-3-kinase (PI3K), which results in its activation [101]. After that, the phosphatidylinositol-3-phosphate interacts with phosphoinositide-dependent protein kinase 1 (PDK1), which phosphorylates protein kinase B (Akt). The phosphorylated Akt then activates mTORC1 via the inhibition of TSC2 [102,103,104]. The activated mTORC1 phosphorylates two molecules: 4E-binding factor 1 (4E-BP1) and ribosomal kinase 70 (p70S6k), which leads to an increase in protein synthesis [105]. It was shown that under disuse conditions IRS-1 is rapidly ubiquitinated by cblb ubiquitin ligase, which lead to its degradation. Cblb expression was prevented by antioxidant administration, such as N-acetylcysteine and TEMPOL, indicating the ROS-induced degradation of IRS [106]. IRS-1 degradation in turn leads to PI3K-dependent signal weakening and Akt dephosphorylation.

In addition to the main pathway, Akt is also capable of phosphorylating and inhibiting glycogen synthase kinase (GSK-3β) at Ser 9, which can lead to an increase in total protein synthesis via translation initiation factor 2B (eIF2B) [107]. GSK-3β phosphorylation at Ser 9 is significantly downregulated as early as after 1 day of rat hindlimb suspension and remains decreased at least during the first two weeks of rat hindlimb unloading [78,83]. Nevertheless, the functioning of the mTOR pathway under functional unloading of the skeletal muscles (in particular, hindlimb unloading) remains unclear. A number of studies have shown an increase in the phosphorylation of p70S6k (which implies an increase in mTOR activity) on days 1–3 of hindlimb unloading [78,83,108]; however, this activation is accompanied by a decrease in the level of protein synthesis. This transitory activation may be caused by inactivity-dependent AMPK downregulation, as AICAR treatment during 1 day of rat HS prevented an increase in p70S6K phosphorylation [80]. At the same time, rapamycin treatment during 1-day HS prevented an increase in the expression nof MuRF-1 and atrogin-1 E3 ubiquitin ligases, so we can conclude that transient mTOR activation contributes to the early development of disuse-induced proteolysis [80].

At the same time, in the later stages of the mechanical unloading of the hindlimbs, as a rule, there is a decrease in the phosphorylation of p70S6k [109,110,111]. Phosphorylation of 4E-BP1, which is also an effector of mTORC1, decreased in the rat soleus muscle in the first days of functional unloading [78,112]. In the soleus muscle of the mouse, it did not change after 3- and 7-day hindlimb unloading [113]. After 14-day hindlimb unloading, according to some authors, the phosphorylation of 4E-BP1 significantly decreased in the rat soleus muscle [109,114], whereas according to other authors, it did not differ from control values [96,115]. Thus, it cannot be ruled out that the contribution of mTOR-dependent signaling pathways to a decrease in protein synthesis in skeletal muscles depends on the stage of functional unloading. The reasons for the nonlinear changes in the activity of mTOR must, apparently, be considered in the context of the fact that mTORC1 receives signals about the energy state of the cell and decreases its activity in case of a deficiency of high-energy phosphates and the activation of AMP-dependent protein kinase (AMPK). A deficiency of ATP and an increase in the level of AMP lead to the activation of AMPK (phosphorylation at Thr172), which is a key energy sensor in the cell. When activated, AMPK can suppress mTORC1 signaling through the phosphorylation and activation of TSC2 [116] or the phosphorylation of Raptor (regulatory-associated protein of mammalian target of rapamycin, a part of the mTORC1 complex), which leads to its subsequent sequestration by regulatory proteins of the 14-3-3 family [117]. The data obtained in our laboratory indicate that AMPK becomes dephosphorylated (at Thr172) in the rat soleus muscle as early as the 1st day after hindlimb unloading [78,118], as well as in the soleus muscle of humans after a 3-day immersion [119], suggesting that the increased activity of mTORC1 at the initial stage of gravitational unloading may be associated with the inactivation of AMPK at this stage. A further increase in AMPK activity (twofold in comparison with the control after 14 days of hindlimb unloading) can lead to the inactivation of mTOR and an increase in atrophy [78].

The molecular mechanisms of mTOR regulation in conditions of the functional unloading of skeletal muscles in humans remain insufficiently clear. In response to unloading, a decrease in the phosphorylation of mTOR and p70S6K was observed after 2 weeks of immobilization [120]. In contrast, other studies did not find a decrease in signal transmission from Akt to mTOR, despite a decrease in protein synthesis [98,121]. It is worth noting that the peak activation of signaling proteins (4E-BP1 and p70S6K) occurs 1–2 h after a meal, whereas muscle biopsies in many studies were taken 3–6 h after a meal, when the response was weakened or possibly absent [122,123,124]. This effect may complicate the analysis of mTOR signaling in humans under disuse conditions.

Another problem related to the unloading-induced changes in protein synthesis is insulin resistance. Insulin resistance (i.e., an impaired metabolic response to endogenous or exogenous insulin) has repeatedly been observed in humans after HDT BR [125,126] and immobilization [127], as well as in rats in a model of hindlimb unloading [128]. Experiments with hindlimb unloading showed that insulin resistance led to a weakening of the Akt/mTORC1 signaling pathway [105] in the soleus and medial gastrocnemius muscles of the rat.

The level of protein synthesis is also influenced by translational capacity, which is characterized by the amount of ribosomes [94]. A decrease in the content of total rRNA, as well as 18S and 28S rRNA, was registered in the soleus muscle after 1, 3, 7 and 14 days of hindlimb unloading in rats [84,110] and after a 21-day “dry” immersion in humans in our laboratory (unpublished data). A decrease in the total concentration of rRNA was also shown in experiments with gravitational muscle unloading in humans [129,130], as well as during periods of inactivity after surgery [131]. Although changes in the content of ribosomes after immobilization were not observed in humans [90,132], the above results indicate that ribosome biogenesis can be disrupted in various variants of functional unloading, contributing to a decrease in protein synthesis [94].

Recent studies in our laboratory showed that nitric oxide plays an important role in the regulation of protein synthesis and GSK-3 activity, as well as the content of ribosomal RNA at later stages of hindlimb unloading (day 7) [85]. Mechanical stimulation of the feet support zones prevented a decrease in the content of 18S and 28S rRNA after 7 days of hindlimb unloading in rats due to nitric oxide production. It also partially prevented a decrease in protein synthesis, which suggests that a decrease in translational capacity under conditions of functional unloading may contribute in a decrease in protein synthesis and is apparently regulated by the level of nitric oxide in the myoplasm of the skeletal muscle [85]. It cannot be ruled out that the NO/GSK-3 (or NO/rRNA) signaling pathway contributes to a decrease in the level of protein synthesis at the earlier stages of hindlimb unloading, including the period of increased mTOR activity. On the 3rd day of the experiment with the mechanical stimulation of the feet support zones, the Akt-independent inactivation of GSK-3 was detected, which is likely to be the result of an increase in NO [81]. NO donor treatment also prevented muscle atrophy after 7 days of mice hindlimb unloading [133]. However, the role of NO in disuse-induced muscle alterations is a subject of a scientific discussion and it will be revealed in detail in the next chapter.

### 3.2. Effect of Reduced Motor Activity on Protein Degradation in Skeletal Muscle

To date, it is impossible to deny the contribution of increased protein degradation to skeletal muscle atrophy in various models of the functional unloading of skeletal muscles [134]. After only 3 days of ULLS, an increase in the content of 3-methylhistidine, a marker of myofibril destruction, in the interfiber space of the vastus lateralis muscle, was registered in the volunteers [135]. Nonetheless, direct measurement of the protein degradation rate, in contrast to measurements of the protein synthesis rate, is rarely carried out. In most studies, conclusions about the rate of protein degradation are made by analyzing the functioning of molecular proteolytic systems.

A number of authors in recent years have turned their attention to the fact that studies on the effects of bed rest in humans showed a significant decrease in the level of protein synthesis and the content of anabolic signaling markers, whereas no changes in the activity and expression of proteolytic enzymes were found [see review [136]]. These authors even suggest the existence of fundamental biological differences between humans and laboratory rodents. For example, the hindlimb unloading model is always accompanied by a high activity of proteolytic systems. However, all arguments in favor of this concept are based on studies in humans on the vastus lateralis muscle, a mixed locomotor muscle with a high proportion of fast-twitch fibers, whereas in studies on laboratory rodents, samples of predominantly slow-twitch postural soleus muscles are used. Even in studies on the vastus lateralis muscle of humans, an increase in the proteolytic enzymes’ expression is observed after a short-term exposure to unloading. Studies of human soleus muscles under conditions of unloading also show increases in proteolytic enzymes [see review [134]].

The main protein degradation systems in skeletal muscle are Са2+-dependent proteases (calpains), the autophagosomal system and the ubiquitin-proteasome system [137]. Calpains promote the degradation of muscle proteins and may be responsible for the destruction of the cytoskeletal structure of myofibrils [138].

The main cellular event that activates calpain is the accumulation of calcium ions in the myoplasm. Such an increase was predicted by G.A. Nasledov [139] and discovered by C. Ingalls [140]. In our laboratory, it was shown that blocking L-type calcium channels in vivo can partially prevent destructive processes in the soleus muscle during unloading [for an overview, see [141]]. The levels of resting cytosolic calcium in the slow-tonic muscles of the hindlimb-unloaded animals were increased on the second day of unloading and remained elevated at least during the next two weeks [140,142]. The overexpression of calpastatin, an endogenous calpain inhibitor, prevented the atrophy of rodent soleus muscles by 30% after 10 days of hindlimb unloading [143]. The administration of the calpain inhibitor prevented the increased ubiquitination of proteins, phosphorylation of FOXO3, and an increase in the expression of ubiquitin ligase MAFbx [144], which indicates the coordinated activation of various proteolytic systems under functional unloading. Moreover, calpain inhibitor administration protects cytoskeletal proteins, including desmin and titin, from degradation, and prevents the unloading-induced decrease in soleus muscle stiffness after 3-days unloading [145]. In recent years, new data indicating a more complex nature of the interaction of calpain-dependent and ubiquitin-proteasome proteolysis in muscle atrophy were obtained. It was recently shown that the intense degradation of desmin, which is characteristic of the initiation stage of denervation atrophy, begins with intense phosphorylation of the substrate by glycogen synthase kinase (GSK3β) and then the ubiquitination of the molecule by the E3 ubiquitin ligase MuRF-1. Only after this does the cytoskeletal protein molecule undergo calpain-dependent fragmentation and processing in the 26S proteasome [146].

Nitric oxide is one of endogenous inhibitors of both calpains and GSK-3, so the lack of NO under conditions of a high resting cytoplasmic calcium level may be one of the causes of the unloading-induced proteolysis upregulation. Moreover, in a recent article we have shown that calpastatin expression in the rat soleus muscle is regulated in an NO-dependent manner [147]. In this study, we have also showed that plantar mechanical stimulation during 7-days with hindlimb suspension prevented the unloading-induced decrease in muscle stiffness as well as the downregulation of desmin, titin and nebulin protein contents in tbe soleus muscles, whereas treatment by nNOS inhibitor L-NAME completely blocked these effects.

However, nNOS activity was shown to trigger the entrance of the FOXO transcription factor into muscle nuclei after 6 h of rat hindlimb unloading in soleus muscle [148], so the role of NO (or more likely the combination of NO and ROS, causing nitrosative stress) during the first 24 h of unloading could seem rather controversial. We previously showed that hindlimb unloading of the rat leads to a significant and profound decrease in the concentration of nitric oxide in the soleus muscle [149,150]. At the same time, Suzuki et al., using a similar model and the same analytical method (EPR spectroscopy), observed a significant increase in the content of nitric oxide in the soleus muscle of mice [151]. These studies differ in a number of technical details. In particular, Suzuki et al. used unfrozen tissue samples, which made it possible for NO synthases to maintain their activity during measurements. A detailed analysis of methodological issues was carried out in the study [150]. These considerations and a number of indirect data (in particular, a profound decrease in the expression of nitric oxide synthase during unloading [148,149,152]) suggest a significant decrease in the level of nitric oxide in the fibers of unloaded soleus muscle. Lomonosova et al. also obtained data indicating the role of this decrease in the activation of the calpain-dependent proteolysis of cytoskeletal proteins: the intensity of their degradation significantly decreased when the animals were administered with the main substrate of nitric oxide synthase L-arginine [149].

Proteolytic fragments produced via the activity of calpains are ubiquitinated and degraded in proteasomes to amino acids by cathepsin peptidases. Much evidence suggests that calpains [153], caspase-3 [154], the autophagolysosomal system of the cell [155] and the ubiquitin-proteasome system [156] are all involved in muscle atrophy caused by unloading. However, under conditions of unloading, the ubiquitin-proteasome system is often regarded as the most important proteolytic system that leads to muscle atrophy [96,137]. For protein cleavage through this system, three different enzymatic components of the ubiquitin-proteasome pathway are required: E1 (ubiquitin-activating enzyme), E2 (ubiquitin-conjugated enzyme) and E3 (ubiquitin ligase, a key enzyme that regulates proteolysis, since it recognizes numerous target protein substrates) [157]. Two muscle-specific E3 ligases (MuRF1 and MAFbx/atrogin-1) play an important role in skeletal muscle atrophy, including that caused by functional unloading [156,158,159]. In various animal models of muscle atrophy under conditions of mechanical/gravitational unloading (hindlimb unloading, immobilization, space flight and denervation), the mRNA levels of both ubiquitin ligases (MuRF1/MAFbx) rapidly increased and supposedly played a decisive role in the initiation of the atrophy process [96,160,161,162,163,164]. Studies using MAFbx- and MuRF1-knockdown mouse models further confirmed the role of these two genes in the development of muscle atrophy. For example, mice deficient in either MAFbx or MuRF1 were found to be more resistant to denervation-induced atrophy [161]. Another study also showed an attenuated atrophy response of the soleus muscle of MuRF1-knockout mice during 10 days of hindlimb unloading [165]. In the studies by our laboratory, the activation of the expression of MuRF-1 and MAFbx was shown to occur already after the first 24 h of the hindlimb unloading of rats [80]; at this stage, the increase in their expression was triggered by the mTOR activation caused by the AMPK inactivation. Moreover, at the conditions of 3-day hindlimb unloading of rats, the administration of the MAP kinase inhibitor p38 was shown to prevent an increase in MuRF-1 expression, as well as the atrophy of the soleus muscles of experimental animals [166]. Inhibition of xanthine oxidase, an activator of the p38 MAP kinase, also prevents an increase in MuRF-1 expression in animals subjected to hindlimb unloading [167].

Apparently, the activation of ubiquitin ligase expression during the functional unloading of skeletal muscles is caused by both the accumulation of high-energy phosphates and the accumulation of reactive oxygen species, which is likely to be triggered, among other factors, by the accumulation of calcium ions in the cytoplasm of muscle fibers. In a recent study, researchers investigated one more calcium-dependent pathway regulating proteolysis under disuse conditions. The “slow” path of Ca2+ accumulation in the myoplasm in between muscle contractions, dependent on ATP/ADP accumulation, their exit from pannexin 1 channels, P2Y receptor activation and PI3/PLC-dependent opening of IP3 calcium channels, was described by E Jaimovich and colleagues [168]. In our laboratory it was shown that the blocking of pannexin 1 channels during 3 days of rat hindlimb suspension results in downregulation of key proteolytic markers MuRF-1 and MaFbx expression, so the described mechanism of calcium accumulation is likely to contribute to the unloading-induced muscle atrophy [82].

Despite the fact that in many works MuRF-1 and MAFbx are considered together, there is evidence that the degree and timing of activation of these two enzymes are different for different muscles, as well as the regulatory mechanisms that determine their expression. Under conditions of unloading, the expression of E3 ubiquitin ligases MuRF1 and MAFbx is known to be regulated by various upstream factors or signaling pathways [156]. For example, in the plantar flexors of the ankle joint (soleus and medial gastrocnemius muscles), more significant and prolonged increases in the expression of the MuRF1/MAFbx genes occurred compared to the dorsi flexor of the foot (tibialis anterior muscle) [160,161]. In our laboratory, the expression of MaFbx, but not MuRF-1, was shown to be regulated by histone deacetylase 1 after 3 days of hindlimb unloading in rats [169]. Mechanical stimulation of the support zones of the feet during 3 days of hindlimb unloading, on the contrary, prevents an increase in the expression of MuRF-1, but not MaFbx, as well as the inhibition of MAP kinase p38 [83].

It should also be noted that there are many studies on human models that show upregulation of MuRF1 and MAFbx during atrophy, caused by unloading [162]. For example, the MuRF1 and MAFbx mRNA content increased significantly after 2-day [170] and 5-day immobilization [18], or after 3-day ULLS [171]. However, in a 20-day bed rest study, an elevated content of MAFbx mRNA, but not MuRF1 mRNA, in the vastus lateralis muscle was observed [172]; the same results were observed during a 14-day immobilization of the legs [173]. On the other hand, an elevated expression of MuRF1 mRNA, but not of MAFbx mRNA, was observed from days 1 to 10 of leg immobilization [121]. Moreover, the MuRF1 protein expression was found to increase in the soleus muscle but not in the vastus lateralis muscle after 60 days of bed rest [174].

The identification of “atrogenes”—genes that are activated independently by atrophy stimulus (denervation, unloading, thermal damage)—attracted much attention from many laboratories [161,173,175]. This led researchers to identify the FOXO transcription factors (FOXO1 and FOXO3) as the main regulators of the expression of both MuRF1 and MAFbx [176,177]. It is noteworthy that the functioning of the FOXO factors is determined by their cellular location; they are regulated via the IGF-1-PI3K-Akt signaling pathway [178,179]. Under normal physiological conditions, Akt phosphorylates FOXO at specific threonine and serine residues, which leads to the retention of FOXO in the cytoplasm instead of translocation to the nucleus [178]. Under conditions of muscle unloading, dephosphorylated FOXO translocates into the nucleus and activates several different types of atrogenes (E3 ligases) or genes associated with autophagy [177]. Several studies reported that levels of FOXO1 or FOXO3 mRNA and protein expression in “slow-twitch” soleus and “fast-twitch” gastrocnemius and plantar muscles are increased in various models of muscle atrophy [163,180,181,182,183]. Sandri et al. found that constitutively active FOXO3 acts on the MAFbx promoter, inducing MAFbx transcription and the severe atrophy of muscle fibers. In addition, they showed that the stimulation of two main proteolytic ubiquitin ligases (MuRF1/MAFbx) through the overexpression of FOXO1 and FOXO3 leads to an additional reduction in muscle mass and strength during atrophy caused by functional unloading [176]. Moreover, direct or indirect inhibition of the transcriptional activity of FOXO and its interaction with other transcription factors results in a decrease in muscle atrophy [184,185,186]. It is interesting that constitutively active FOXO3 also controls the stimulation of the autophagic/lysosomal proteolysis pathway, thus leading to muscle atrophy in fasting and denervation models [187].

The third mechanism of proteolysis in skeletal muscle is autophagy. Autophagy is responsible for the removal of unfolded, damaged and dysfunctional proteins and organelles through the formation of autophagosomes for degradation by lysosomes [155]. By the 24th day of bed rest, the activation of autophagy markers such as beclin-1 was detected, suggesting an increase in the formation of autophagosomes and therefore a higher activity of macroautophagosomes (autophagosomes for cleaving organelles); however, other markers of autophagy, such as P62, the ratio of LC3II/I and cathepsin-L, were not activated [12].

**Figure 2:**
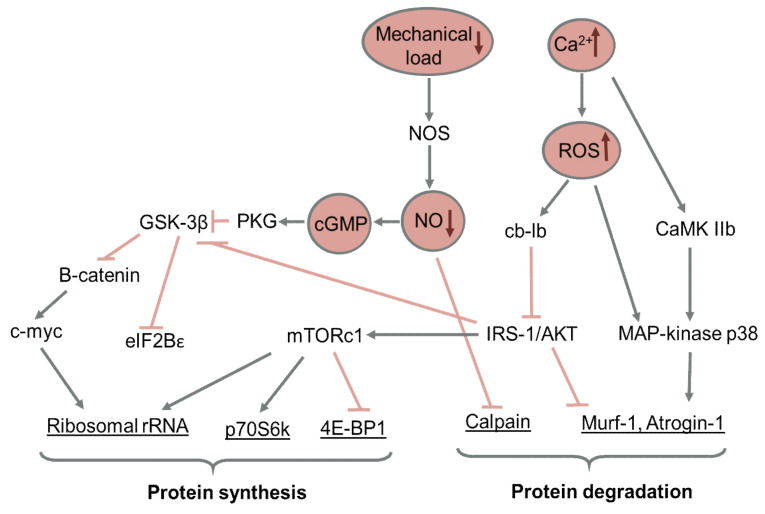
Key signaling pathways regulating protein turnover during muscle disuse. Pointed arrows show activation; blocked arrows show inhibition.

A scheme summarizing the key signaling pathways regulating protein turnover during muscle disuse is presented in Figure 2.

### 3.3. Effect of Reduced Activity on Changes in Muscle Fiber Types

As mentioned above, skeletal muscle fibers are divided into two types, “fast-twitch” and “slow-twitch”. “Fast-twitch” muscle fibers demonstrate a high strength and speed of contraction, along with rapid fatigability. “Slow-twitch” muscle fibers show low fatigability and a relatively low maximum strength and speed of contraction. The properties of muscle fibers are determined by the predominance of one or another isoform of myosin heavy chains (MyHCs) in the fibers. In the skeletal muscle of mammals, there are four main isoforms of MyHCs: the “slow-twitch” isoform MyHC I (β) and three “fast-twitch” isoforms IIa, IId/x- and IIb- [45]. In humans, the MyHC isoform IIb is absent [45]. Functional unloading of skeletal muscles caused by the absence of contractile activity leads to a shift in the myosin phenotype, i.e., slow-twitch MyHC isoform expression decreases and fast-twitch isoform expression increases. This slow-to-fast fiber-type shift has been observed as early as after 1 week of “dry” immersion [188]. In astronauts, a decrease in the proportion of slow-twitch skeletal muscle fibers was observed after a 6-month space flight [189] and an 11-day flight [190]. The transformation of the myosin phenotype to the “fast-twitch” type was also observed in volunteers after 84-day bed rest [10] and 35-day bed rest [191]. To the greatest extent, the slow-to-fast transformation of muscle fibers is manifested when the spinal cord is damaged [192].

In an animal model of gravitational unloading, mRNA of the slow-twitch MyHC isoform was observed in the soleus muscle already after the 1st day of hindlimb unloading [118,193]. Moreover, after the 1st day of hindlimb unloading of the rodents, the expression of mRNA of the “fast-twitch” MyHC isoforms IId/x and IIb increased in the soleus muscle and remained elevated for at least 28 days of hindlimb unloading [193,194]. After the 7th day of hindlimb unloading and later, numerous studies showed a significant decrease in the expression of the slow-twitch MyHC isoform both at the mRNA and protein levels [149,150,193,194,195]. From day 3 to the end of the 1st week of hindlimb unloading, the expression of MyHC IIa mRNA was decreased, but after that its expression began to increase and after 14 days of hindlimb unloading it was as high as the control values [194,196].

Studies of the reasons for the change in the expression pattern of myosin genes in the conditions of functional unloading have been carried out mainly on models of the hindlimb unloading of rodents. A key role in the decrease of the slow-twitch MyHC isoform during hindlimb unloading is played by the AMPK/HDAC4 and NO/GSK-3/NFATc1 signaling pathways. Both of these signaling pathways are activated when a muscle is activated The AMP-activated protein kinase (AMPK) is activated upon the consumption of ATP in muscle and the accumulation of ADP and especially AMP, as well as upon a decrease in glycogen content in the muscle; activated AMPK phosphorylates histone deacetylase 4 (HDAC4) is the slow-twitch MyHC transcriptional repressor, and removes HDAC4 from the muscle nuclei. The administration of an AMPK activator AICAR on the 1st day of hindlimb unloading prevents both the accumulation of histone deacetylase 4 in the muscle nuclei of the soleus muscle and a decrease in the expression of mRNA of the slow-twitch MyHC isoform [118]. The application of β-guanidine propionic acid, an activator of AMPK which decreases the ATP/ADP+AMP ratio, prevents the slow-to-fast transformation of the myosin phenotype of soleus muscles after 10 days of hindlimb unloading [197].

The second pathway regulating the expression of slow-twitch MyHC fibers under the conditions of functional unloading is the NO/GSK-3/NFATc1 pathway. NFATc1 (nuclear factor of activated T-cells, cytoplasmic 1) is a transcription factor that, when in the nucleus, binds to the promoter of the gene encoding the slow-twitch MyHC isoform and activates its expression. NFATc1 can be phosphorylated and thereby removed from the nucleus by protein kinases such as GSK-3β, MAP kinase p38 and JNK [198,199]. Calcium-dependent phosphatase calcineurin dephosphorylates NFATc1, which leads to its return to the muscle nuclei and the restoration of its ability to activate the expression of the gene encoding the slow-twitch MyHC isoform.

In experiments with fluorescent reporters of the transcriptional activity of NFATc1 in vivo in skeletal muscles of rats, NFATc1 was shown to be a sensor of neuromuscular activity of skeletal muscles: the level of NFATc1-dependent transcription is higher in slow-twitch muscles than in fast-twitch ones, whereas in denervated slow-twitch muscles its transcriptional activity decreases, but after a low-frequency electrical stimulation of denervated muscles it is restored [200].

The content of NFATc1 in muscle nuclei, as well as its transcriptional activity, decrease already on the 1st day of hindlimb unloading, which is accompanied by GSK-3β kinase activation [81]. By the 7th day of hindlimb unloading, the content of NFATc1 in the muscle nuclei and its transcriptional activity are also reduced; the pharmacological blocking of GSK-3β kinase or an increase in the level of nitric oxide in the muscles both lead to the restoration of the NFATc1 content in the muscle nuclei and to the restoration of slow-twitch MyHC isoform expression [150,201]. It should be noted that mechanical stimulation of the support zones of the feet restores the relative proportion of fast-twitch and slow-twitch fibers and leads to the restoration of NFATc1 myonuclear content and its transcriptional activity, and this effect depends on the activity of the NO synthase [152]. The MAP kinase p38 also contributes to NFAT-dependent transcriptional activity and to the expression of the slow-twitch MyHC isoform during the hindlimb unloading of rats [202].

### 3.4. Effect of Reduced Muscle Activity on Muscle Oxidative Capacity

Aerobic performance is the ability to perform long-term work (several minutes/tens of minutes), the energy supply of which is mainly provided due to oxidative reactions. Aerobic performance depends on the ability of the cardiovascular system to deliver O2 to skeletal muscles and on the ability of muscles to utilize the O2 [203]. In untrained people and people with reduced muscle functional capacities, aerobic performance is limited first of all by the low oxidative capacity of skeletal muscles and their high fatigability, rather than by the oxygen delivery [204].

Muscle capillary density is one of the key parameters affecting the diffusion of O2 to the mitochondria of muscle fibers [203]. Most studies have shown a decrease in the number of capillaries per fiber [174,205,206] and capillary density, [207,208,209,210] in the vastus lateralis muscle in humans after several weeks of restricted physical activity, whereas other studies did not detect any changes in these parameters [211]. In accordance with these data, three weeks of one-leg immobilization caused multidirectional changes in the expression of genes associated with the regulation of angiogenesis in the vastus lateralis muscle; less than half of them (including the key regulators of angiogenesis VEGFA and VEGFB) decreased their expression, whereas the remaining genes’ expression increased [212]. Numerous studies have convincingly shown that in human muscles, a decrease in motor activity is accompanied by a decrease in the density of mitochondria and the maximum respiration rate, which negatively affects the maximum rate of oxidation of fats and carbohydrates and reduces aerobic performance at the level of the whole organism (see reviews [213,214]). Two-dimensional gel electrophoresis and mass spectrometric analysis made it possible to study changes in the content of several tens of muscle proteins. So, after 8 and 35 days, as well as for longer periods (55 and 65 days), of bed rest in the vastus lateralis muscle, a similar decrease in the content of several enzymes of the Krebs cycle and oxidative phosphorylation was observed [12,215,216]. That is, with a decrease in motor activity, the oxidative capacities of skeletal muscles rapidly decrease and then are kept at the achieved level. It is interesting to note that after two days of leg immobilization in the vastus lateralis muscle, the expression of some genes encoding mitochondrial proteins decreases [170]. With an increase in the duration of hypokinesia, the expression of several tens or even hundreds of genes encoding mitochondrial proteins decreases—these include enzymes of the tricarboxylic acid cycle, electron transport chain, ubiquinone biosynthesis, as well as beta-oxidation enzymes (HADH, ACADL, ACADM, ACADVL) and fatty acid transporters (CD36) [170,217,218,219,220,221,222].

The regulation of fiber metabolism is closely related to the slow-to-fast fiber-type transformation. In general, slow-twitch fibers in humans contain a greater number of mitochondria and the content and activity of respiratory enzymes than fast-twitch fibers. Under various types of unloading conditions mitochondria undergo alterations in function (including a decreased respiratory rate and increase in ROS production and a decline in mitochondrial biogenesis and in activated mytophagy) [223]. At the same time, the markers of mitochondrial fusion and fission are both downregulated, but the changes in fusion markers are usually more profound than in fission markers, thereby activating mitochondrial fission [223]. Alterations of mitochondrial functioning precede the downregulation of mitochondrial biogenesis, occurring as early as after 24 h of muscle immobilization [224]. A number of reviews describe the causes of the increased ROS production by mitochondria in the unloaded muscles, and among the main causes of these alterations are increases in myoplasmic and intramitochondrial calcium levels [223,225]. However, there is a lack of experimental data directly showing the mechanisms connecting unloading-induced cytoplasmic calcium accumulation and the production of ROS by mitochondria. Moreover, the regulation of mitochondrial functioning, dynamics and biogenesis may be controlled by different signaling mechanisms, although all these parameters contribute to muscle oxidative capacity. Some evidence shows that calpain activation, as well as an increase in the ATP/ADP ratio, also contribute to alterations in mitochondrial functions under various disuse conditions [197,226]. Many authors suggest that mitochondrial alterations, especially PGC-1α inactivation, induced by muscle inactivity are the main cause of the unloading-induced muscle atrophy [113,227]; however, we did not detect any countermeasure effect of PGC-1α expression decrease prevention on soleus muscle weight in the hindlimb-unloading experiments conducted in our laboratory [150]. However, the most studied pathway regulating mitochondrial oxidative capacity under disuse conditions is still the PGC-1α-dependent pathway.

In humans, the content of enzymes of the mitochondrial respiratory chain in the vastus lateralis muscle decreases already after 10 days of bed rest [228]; a decrease in the expression of the key regulator of mitochondrial biogenesis, PGC-1α, is also observed [229]. A decrease in PGC-1α expression and the content of the mitochondrial respiratory chain enzymes, along with the disruption of mitochondrial functions, are observed in the muscles of patients in intensive care; however, it is not known whether this decrease is caused by the functional unloading of the muscles or concomitant factors [207,230]. By the 3rd day of hindlimb unloading, the content of PGC-1α in the soleus muscle of rats significantly decreases [83]. Both after 7 and 14 days of hindlimb unloading, the expression and content of PGC-1α in the soleus muscles remains decreased, as does the content of enzymes of the mitochondrial respiratory chain [150,231,232]. Studies on transgenic animals show that PGC-1α is closely related to muscle fatigue resistance [233,234]. Thus, a decrease in the expression of PGC-1α, along with the slow-to-fast transformation of skeletal muscle fibers, can be the cause of increased muscle fatigue during functional unloading.

**Figure 3:**
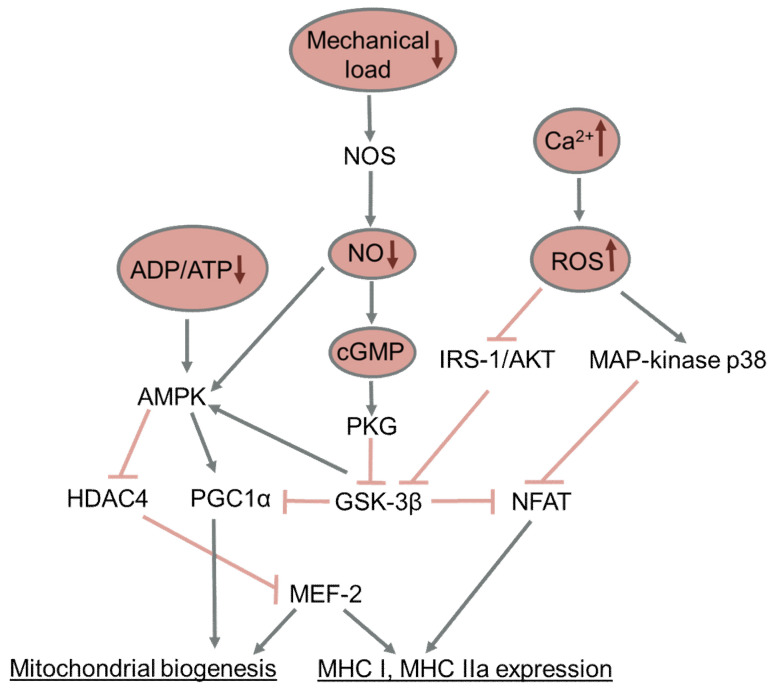
Key signaling pathways regulating slow-to-fast fiber-type transformation and mitochondrial biogenesis during muscle disuse. Pointed arrows show activation; blocked arrows show inhibition.

Little is known about the molecular mechanisms of the decreased expression of PGC-1α under the conditions of functional unloading. Inhibition of GSK-3β during 2-week hindlimb unloading was shown to prevent a decrease in the content of PGC-1α and components of the mitochondrial respiratory chain [231]. The administration of nitric oxide donors during hindlimb unloading and mechanical stimulation of the support zones of the feet both prevented the activation of GSK-3β and decreased the expression of PGC-1α, along with the prevention of a decrease in the expression of the slow-twitch MyHC isoform [83,150]. It was shown on myotubes C2C12 that PGC-1α expression can be regulated via the action of GSK-3β on the TFEB transcription factor [235]. It was also shown that the slow-twitch MyHC isoform can regulate the expression of PGC-1α by means of micro-RNA-499 [236]. Thus, GSK-3β kinase is a key regulator linking the activity of skeletal muscle and the slow-twitch, oxidative phenotype of muscle fibers, which determines muscle resistance to fatigue. The key signaling pathways affecting slow-to-fast fiber-type transformation and mitochondrial biogenesis are summarized in Figure 3.

## 4. Countermeasures to Changes Caused by Reduced Motor Activity

As can be seen from the above, the several negative effects of chronic decrease on the contractile activity of the muscles have a dramatic effect on their work capacity and lead to profound systemic changes that threaten human health and quality of life. It is clear that it is necessary to use a complex of measures to prevent the negative consequences of complete or partial muscle inactivation. Great progress in the development of such a complex has been achieved in space medicine.

Based on the results of studies carried out for the medical support of space flights, as well as international studies of the effects of microgravity on the musculoskeletal system of humans and animals, Professor I.B. Kozlovskaya developed a number of theoretical generalizations that made it possible to systematically analyze the effects of microgravity and create the appropriate countermeasures. According to her theory, the key effect of microgravity on the musculoskeletal system is the elimination of support afferentation [23]. The elimination of support afferentation leads to inactivation of the postural muscles motor units, which in turn leads to atrophy, atony, transformation of the myosin phenotype and subsequent functional disorders of skeletal muscles [23,237]. At the same time, the mechanical loading of skeletal muscles also contributes to atrophy of both slow-twitch and fast-twitch muscle fibers [1,238]. Countermeasures against the disorders caused by muscle disuse are based on providing support afferentation and/or mechanical loads for muscles in one way or another. To provide a mechanical load, it is recommended to use various exercise systems and devices that provide an axial load (which are suited to providing a load on the muscles and the force of acceleration in centrifuges) [239]. For the restoration of support afferentation and, accordingly, muscle activity, there are devices that stimulate the mechanoreceptors of the feet, which lead to neuromuscular activation [240,241]. Electrical stimulation makes it possible to directly activate fast-twitch and slow-twitch motor units in accordance with the impulse patterns specific to a particular type of muscle fibers and thus activate the muscle in the absence of load, which provides an effect similar to the restoration of support afferentation [25]. In recent years, methods of pharmacological countermeasures against the effects of microgravity are also developing, which make it possible to reproduce the effects of muscle activity and/or loading on various intramuscular molecular pathways, but these methods are still rarely used in clinical practice [150,231,242]. A classification of countermeasure methods according to the sensory system they affect is listed in Table 2.

Countermeasures against functional and structural changes in skeletal muscles caused by gravitational unloading play an important role in the medical support of manned space flights. Numerous studies conducted with the participation of cosmonauts and astronauts on the orbital stations Salyut, Mir and the International Space Station, as well as under the simulated microgravity conditions on Earth, show that the changes caused by microgravity can be partially or completely prevented by means of countermeasures [240,241,243,244].

In the 2010s, the “Profilaktika” experiment was carried out on the ISS with the participation of 15 Russian cosmonauts. Within the framework of this experiment, two protocols of countermeasures were compared—intensive interval exercise combining aerobic and anaerobic loading zones, and prolonged low intensive aerobic exercise. Interval exercise in space flight led to the preservation of muscle function almost at the pre-flight level [243]. In 2020, the effectiveness of high-intensity exercise on orbit was confirmed in an experiment by NASA [244].

The workout system of Russian cosmonauts in orbit includes two 1-h sessions during the day, with three days of exercise, followed by one day of rest. The exercise includes treadmill running and bicycle ergometer cycling, with the energy cost ranging from 380 to 600 kcal per day [245]. Since not all cosmonauts perform the prescribed exercises in full throughout the entire flight, the most informative studies are those of the effectiveness of the countermeasures against microgravity-induced disorders in terrestrial conditions, when the effects of microgravity are simulated using HDT BR, unilateral lower limb suspension and “dry” immersion.

Intense exercise on a bicycle ergometer was not found to be efficient in preventing changes caused by functional unloading in the lower leg muscles [246]. At the same time, high-intensity jumping exercises prevented a decrease in maximum muscle strength and in muscle mass after 60-day HDT BR [208], whereas resistive exercises prevented atrophy of vastus lateralis muscle fibers after a 14-day bed rest [29], which correlates well with data indicating the higher efficiency of high-intensity workouts under weightlessness conditions. Nevertheless, high-intensity jumping exercises during a 60-day HDT BR had a rather weak countermeasure effect on changes in the myosin phenotype of fibers and the decrease in the oxidative capacity of the lower limbs’ skeletal muscles [209].

In addition to exercise, there are other methods of increasing the level of muscles’ mechanical loading. The use of the “Penguin” axial loading suit during a 2-month and 4-month HDT BR prevented the atrophy of the slow-twitch soleus muscle fibers [247]. Vibration stimulation of the plantar fascia during rat hindlimb unloading or cast immobilization leads to a partial prevention of muscle fiber atrophy, but does not prevent the slow-to-fast fiber-type transformation [210,248]. In 2019, vibration stimulation of the plantar fascia was shown to activate the mechano-dependent regulators YAP1 and ERK [248], which suggests that the effects of vibration stimulation are caused not only by the activation of sole proprioceptors and the restoration of muscle activity, but also via the activation of muscle mechanosensors and by triggering signaling cascades that respond to loading. The use of massage as a method of activating muscle mechanoreceptors increased the level of protein metabolism in the gastrocnemius muscle of rats during 7 days of hindlimb unloading, but did not lead to the atrophy prevention or a decrease in the rate of degradation of cytoskeletal proteins [249].

It some studies, it was shown that reductions in skeletal muscle mass and functional properties could be prevented through the application of artificial gravity via centrifugation. The use of artificial gravity by rotation of the test subjects in a centrifuge with an acceleration of 2.5 G in the area of the feet for one hour per day prevented a decrease in protein synthesis after 21-day HDT BR, as well as soleus and vastus lateralis atrophy and the “fast-twitch” transformation of soleus muscle fibers [250,251]. Another work shows that intermittent centrifugation can partially maintain the mass of the rat soleus muscle during unloading [252]. After a 5-day HDBR, there was a positive effect on the maximum strength of leg muscle contractions and the height of jumps in groups with a 30-min centrifugation in a short-radius centrifuge, as well as with six 5-min centrifugation sessions [253]. In the experiments with rodents, it was shown that 30-day 2G centrifugation induces a distinct anabolic response in mouse soleus and tibialis anterior muscles [254]. Unfortunately, there are very few studies on the effect of artificial gravity as a countermeasure against changes caused by functional unloading.

Another approach to countermeasures is the restoration of support afferentation, which leads to the activation of skeletal muscles’ motor units (that is, the muscle fiber and the motor neuron that innervates it). The effects of support afferentation on the state of the human musculoskeletal system were first shown in the 1983 Soviet–Cuban experiment during a 7-day space flight [32]. In a subsequent experimental series using the “dry” immersion model, it was found that the use of mechanical stimulation of the support zones of the feet allows the maintenance of a normal level of electrical activity and reflex tonus of the skeletal muscle [188]. In this experimental series, a pressure of 40 kPa was applied to a foot daily during 6-h sessions, with a regimen of a 20-min stimulation, followed by a 40-min rest period. The stimulation comprised two modes, corresponding to slow walking (75 steps per min) and fast walking (120 steps per min), with each mode implemented for 10 min. Mechanical stimulation of the support zones of the feet prevented the atrophy of soleus muscle slow-twitch fibers, as well as slow-to-fast fiber-type transformation after a 7-day “dry” immersion and partially prevented the degradation of cytoskeletal proteins [26,31,255]. Studies in rats with mechanical stimulation of the feet support zones during hindlimb unloading showed that the restoration of support afferentation prevents a change in the pattern of myosin gene expression, a decrease in the level of PGC-1α expression and protein synthesis, an increase in ubiquitin ligase expression under conditions of 1 and 3 days of functional unloading, as well as preventing the atrophy of the soleus muscle [81,83,256]. Recent studies show that a key role in most of the countermeasure effects of support stimulation is played by the activity of neuronal NO synthase [85,152].

An effect comparable to the restoration of support afferentation for slow-twitch skeletal fibers is exerted by electrical stimulation of skeletal muscle. Electrical stimulation is based on the restoration of the normal electrical activity of muscles using electric currents with characteristics similar to the patterns of fast-twitch (50–60 Hz) or slow-twitch (10 Hz) innervation. In sports medicine, however, higher-frequency stimulation patterns are used (up to 100 Hz) [257]. Electrical stimulation is employed both directly (electrodes are applied to the skin or to the muscle and the current directly impacts the muscle) and indirectly (by stimulating the nerve that innervates the muscle). Electrical stimulation leads to the activation of muscle fibers and thus allows one to maintain muscle activity. The use of electrical stimulation as a countermeasure against changes caused by functional unloading is the only possible measure for patients who for some reason cannot perform physical exercises, for example, for those who are unconscious. A randomized study showed the effectiveness of high-frequency (45 Hz) electrical stimulation in the prevention of quadriceps femoris atrophy of patients in intensive care [258]. High-frequency stimulation (50 Hz) led to hypertrophy of “fast-twitch” muscle fibers in the vastus lateralis muscle in cancer patients undergoing chemotherapy, but did not prevent a reduction in the number of mitochondria [259]. In sports medicine, electrical stimulation is often used to help an athlete to recover after injuries, after surgery on the ligaments or during cast immobilization. The use of stimulation with a frequency of 50 Hz in athletes after surgery for the restoration of the anterior cruciate knee ligament led to the partial prevention of a decrease in quadriceps strength, but did not prevent the atrophy of the studied muscle [260]. Sessions of electrical stimulation with a frequency of 30 Hz throughout 40 days of leg immobilization due to a fracture of the lower leg led to prevention of a decrease in the level of protein synthesis and atrophy of the quadriceps in comparison with the contralateral leg [132]. High-frequency stimulation also prevented an increase in the expression of ubiquitin ligases MuRF-1, MaFbx and myostatin during a 4-day leg immobilization in the knee joint [18].

Low-frequency electrical stimulation is used in the rehabilitation of patients with chronic systemic damage of skeletal muscles, for example, as a result of spinal cord injury. Unlike high-frequency stimulation, low-frequency stimulation primarily affects the mitochondrial enzyme content and oxidative capacity of the muscle, as well as muscle resistance to fatigue. Studies in rodents conducted in the 1980s showed that chronic low-frequency stimulation leads to the fast-to-slow fiber-type transformation, as well as increases in capillarization, the level of mitochondrial enzymes and muscle resistance to fatigue [261]. An increase in resistance to fatigue with low-frequency stimulation was also observed in the tibialis anterior muscle in humans [262].

Low-frequency electrical stimulation (2–10 Hz) leads to an increase in the expression of the key factor of mitochondrial biogenesis, PGC-1α, in the vastus lateralis muscle of paralyzed patients with spinal cord injury 3 h after the end of the stimulation session [263]. In patients with chronic heart failure, low-frequency (15 Hz) electrical stimulation led to an increase in maximum oxygen consumption (VO2), an increase in citrate synthase activity, and transformation of the vastus lateralis muscle fibers to the “slow-twitch” fiber type [264].

In the 1970s, Soviet scientists carried out studies on the effects of electrical stimulation during prolonged bed rest. For instance, in two series of studies the efficacy of electrostimulation training was assessed using a “Tonus-2” apparatus [265]. In the first series, two different modes of electromyostimulation were used on two groups of volunteers (each consisting of four people) subjected to 49-day bed rest, whereas the volunteers of the third (control) group were under conditions of bed rest without additional influences. The volunteers of the control group had a significant decrease in the cross-sectional area of the muscle fibers of all types in soleus muscle. The size of the muscle fibers of the volunteers subjected to electromyostimulation did not change. Currently, Russian cosmonauts in orbit use the Tonus-3 electrostimulator for stimulation of the quadriceps, abdominal muscles and muscles of the neck, back and shoulders at a frequency of 60 Hz and Stimul-01-LF for muscle stimulation at a frequency of 50 to 25 Hz, depending on the operating mode [241].

However, the use of low-frequency stimulation as a countermeasure against changes caused by functional unloading has certain disadvantages; in particular, the use of chronic 15 Hz stimulation in healthy volunteers may decrease the maximum voluntary strength of knee extensors and the size of muscle fibers [266]. Nevertheless, electrical stimulation in combination with muscle stretching in this experiment prevented the decreases in the size of the fibers and muscle strength.

Studies of changes in molecular regulatory mechanisms during the electrical stimulation of skeletal muscles have mainly been performed on the model of rodent hindlimb unloading and on cultures of myoblasts. Among the triggering mechanisms determining the response of muscle fibers to low-frequency electrical stimulation, various authors mention a decrease in the ATP/ADP ratio, which leads to the activation of AMP-activated protein kinase and calcium ion accumulation, activating the calcineurin/NFATc1 signaling pathway in the myoplasm of skeletal muscle fibers [267,268,269]. The effect of low-frequency electrical stimulation on soleus muscle atrophy during rat hindlimb unloading differs depending on the mode of electrical stimulation. A study with 8-h stimulation reported the partial prevention of atrophy and a decrease in the stiffness of the rat soleus muscle [270], whereas studies with continuous electrical stimulation did not find any prevention of atrophy [109,271]. At the same time, the prevention of the slow-to-fast fiber-type transformation was observed in all three of the above studies. However, the poor effect of continuous electrical stimulation is rather predictable, as it is well-known that the processes of protein synthesis in skeletal muscles are activated during the periods of rest when muscle does not contract.

The least-studied type of countermeasures against disorders caused by muscle functional unloading is the use of various pharmaceutical drugs and food additives. Attempts to prevent muscle atrophy under conditions of bed rest with amino acid or protein supplements have showed contradictory results. Some researchers believe that the use of protein supplements in excess of the recommended intake of 1 g of protein per 1 kg of body weight per day does not lead to the prevention of unloading-induced atrophy. Accordingly, in those studies which detected a positive effect of protein supplements on muscle state, it was caused by replenishing the protein deficiency in the diet of test subjects [272]. Nevertheless, it was shown that the use of amino acid supplements during immobilization and bed rest has a positive effect on protein synthesis, but only partially prevents atrophy [15,273]. The supplementation of food with essential amino acids partially prevents atrophy during bed rest [273,274]. The combination of resistive exercise and the supplementation of food with essential amino acids during a 28-day bed rest effectively prevented a decrease in the mass and strength of the thigh muscles of test subjects [275]. Interestingly, testosterone treatment (especially in combination with exercise) is effective to prevent decreases in lean body mass and muscle strength, aerobic performance and the muscle content of some structural and mitochondrial proteins under conditions of 70-day bed rest. The literature on the use of antioxidants as countermeasure food additives against changes in skeletal muscles under functional unloading is also very controversial. Despite the fact that an increase in the level of reactive oxygen species in various forms of functional unloading has been shown in numerous studies, the main source of reactive oxygen species during unloading has not yet been reliably identified [276]. Antioxidants such as vitamin E, curcumin, astaxanthin and green tea extract have shown both the partial prevention of atrophy during immobilization or hindlimb unloading and the absence of any effect, depending on the dose of the antioxidant and the stage and model of functional unloading [277,278,279].

In recent years, research has begun on pharmaceutical drugs that modulate various molecular signaling pathways involved in the muscle response to functional unloading. The use of such drugs in humans is limited, since it is necessary for these substances to be preliminarily tested for safety. However, there are a number of promising pharmaceuticals already in clinical use in other fields that are also capable of preventing some of the effects of functional unloading. These pharmaceuticals include the p38 MAP kinase inhibitor; its use during 3-day hindlimb unloading in rats led both to the prevention of atrophy and to the prevention of a decrease in the expression of PGC-1α and mRNA of the slow-twitch myosin isoform [166,202]. The use of GSK-3β kinase inhibitors during hindlimb unloading in rats led to the prevention of a decrease in the expression of PGC-1α and mitochondrial proteins and the prevention of the slow-to-fast fiber-type transformation [201,231]. In recent years, GSK-3β was shown to play a significant role in the development of changes in postural muscles during variants of muscle unloading; the inhibition of GSK-3 can lead to an increase in the work capacity of skeletal muscles in rodents, which makes GSK-3β inhibitors some of the most promising pharmacological agents as a countermeasure against unloading-induced changes [280,281].

## 5. Conclusions

The functional unloading of skeletal muscles occurs both in the conditions of space flight and, in one form or another, in terrestrial conditions. To date, all the forms of the functional unloading of skeletal muscles are known to result in muscle atrophy, and the transformation of fibers from the “slow-twitch” type to the “fast-twitch” type, which leads to a decrease in the maximum strength and speed of muscle contraction, as well as to a decrease in their work capacity. The signaling mechanisms of these changes, as well as the triggers that cause them, are far from fully known; there are many questions that still remain open, e.g., about the relationship between the role of muscle electrical activity and its mechanical loading in the development of changes caused by unloading, about signaling mechanisms that initiate a decrease in protein synthesis and an increase in its degradation, as well as a decrease in the oxidative capacity of skeletal muscles. The use of muscle electrical stimulation and mechanical stimulation of the support zones of the feet allows us to prevent a number of negative changes caused by functional unloading, but only in recent years is it beginning to become clear through which molecular mechanisms these countermeasures work. To date, however, enough data have been accumulated that it is now possible to commence the development of pharmacological methods for the correction of changes caused by functional unloading by targeting the signaling pathways that control protein synthesis and degradation, as well as the myosin phenotype and mitochondrial biogenesis in skeletal muscles. Further studies of the molecular mechanisms that trigger skeletal muscle changes occurring during unloading will allow us to expand the range of possible pharmacological targets and physiotherapeutic methods, as well as to improve existing methods and maintain human health in conditions of reduced or completely absent skeletal muscle activity.

## Figures and Tables

**Table 1:** Classification of the types of in vivo inactivation of skeletal muscles.

	Slow-Type Fibers		Fast-Type Fibers	
**Inactivation of Muscles of Systemic Genesis**	**CSA Decrease**	**Force Decrease**	**CSA Decrease**	**Force Decrease**
A. With limitation or elimination of anti-gravity component				
a. With complete or almost complete elimination of anti-gravity component				
- Space flight/Kepler parabola flight	**+++**	**+++**	**++**	**++**
- Immersion	**+++**	**+++**	**++**	**++**
- Unloading of all limbs or only hindlimbs of rats	**+++**	**+++**	**++**	**++**
- Spinal transection or spinal isolation	**++++**	**++++**	**++++**	**++++**
- Systemic muscle inactivation with artificial life support (“intensive care unit” model)	**++++**	**++++**	**++++**	**++++**
b. With partial elimination or limitation of anti-gravity component				
- Unilateral lower limb suspension	**+++**	**+++**	**++**	**++**
- Bed rest	**++**	**++**	**++**	**++**
B. Without limitation or elimination of anti-gravity component				
- Restriction of locomotor activity	**-**	**-**	**++**	**++**
Inactivation of muscles of local genesis				
- Immobilization of joint with cast	**++**	**++**	**++**	**++**
- Denervation	**+++**	**+++**	**++**	**++**
- Tenotomy	**+++**	**+++**	**++**	**++**
- Inactivation of diaphragm by forced ventilation	**++++**	**++++**	**++++**	**++++**

**Table 2:** Main countermeasure methods used to prevent unloading-induced muscle alterations, and the sensory structures that respond to them.

Countermeasure	Sensory Structures
Exercise	whole body
Plantar stimulation	plantar mechanoreceptors
Neuromuscular electrostimulation	motor nerve voltage-operated channels
Muscular electrostimulation	muscle voltage-operated channels
Axial load	mechanosensitive structures of muscles, tendons and bones
Centrifuge	mechanosensitive structures of muscles, tendons and bones; vestibular system
Vibration stimulation	mechanosensitive structures of muscles, tendons and bones

## Abbreviations

4E-BP1	eukaryotic initiation factor 4E binding protein
AKT	protein kinase B
AMPK	AMP-activated protein kinase
cGMP	cyclic guanosine monophosphate
c-Myc	c-myelocytomatosis oncogene (transcription factor)
CSA	cross-sectional area
eIF2B	eukaryotic initiation factor 2B
FoxO	forkhead box O protein
GS1	glycogen synthase-1
GSK-3β	glycogen synthase kinase-3β
HDT BR	head down tilt bed rest
HS	hindlimb suspension
HU	hindlimb unloading
IGF-1	insulin-like growth factor 1
L-NAME	N(gamma)-nitro-L-arginine methyl ester
MAFbx	muscle atrophy F-box protein/atrogin 1
MAPK	mitogen-activated protein kinase
MCIP 1.4	modulatory calcineurin-interaction protein 1.4
MEF-2	myocyte enhancer factor 2
mir-208	micro-RNA 208
mTORC1	mammalian/mechanistic target of rapamycin complex 1
MuRF1	muscle RING finger protein
MyHC	myosin heavy chain
NFAT	nuclear factor of activated T-cells
NO	nitric oxide
NRF	nuclear respiratory factor
p70S6K	ribosomal protein S6 kinase p70
PGC-1α	peroxisome proliferator-activated receptor gamma coactivator 1-alpha
PI3K	phosphatidylinositol 3-kinase
PKA	protein kinase A
PKB	protein kinase B (AKT)
PKC	protein kinase C
PKD1	protein kinase D1
PKG	protein kinase G
PMS	plantar mechanical stimulation
PuRA	purine Rich Element Binding Protein A
PuRB	purine Rich Element Binding Protein B
rRNA	ribosomal RNA
SOX-6	transcription factor
TFAM	mitochondrial transcription factor A
